# Thrombin-antithrombin complex measurement using a point-of-care testing device for diagnosis of disseminated intravascular coagulation in dogs

**DOI:** 10.1371/journal.pone.0205511

**Published:** 2018-10-10

**Authors:** Kenji Rimpo, Aki Tanaka, Masayasu Ukai, Yuichi Ishikawa, Miyuki Hirabayashi, Toshihiro Shoyama

**Affiliations:** 1 Saitama Animal Medical Center, Iruma-shi, Saitama, Japan; 2 Department of Wildlife Medicine, Nippon Veterinary and Life Science University, Musashino-shi, Tokyo, Japan; Duke University School of Medicine, UNITED STATES

## Abstract

Reference interval for thrombin-antithrombin complex (TAT) level was determined using an in-house TAT measurement device, and its validity for diagnosis of disseminated intravascular coagulation (DIC) was evaluated in dogs. One hundred and two clinically healthy dogs and 247 diseased dogs with conditions that potentially caused DIC were recruited in the study. Six diagnostic testing for DIC were evaluated in diseased dogs and the diseased dogs were categorized into five groups depending on abnormal findings. TAT was measured in all study animals and between-group differences were evaluated. TAT level was positively associated with severity of DIC. There were no significant differences in TAT levels among clinically healthy dogs, diseased dogs without any abnormal finding and diseased dogs with one abnormal finding in the DIC diagnostic testing. TAT levels in groups with two or more abnormal findings were significantly higher than clinically healthy dogs. Reference interval of TAT level for clinically healthy dogs was ≤ 0.25 ng/ml. Validity of using TAT for early detection of DIC was evaluated. In-house TAT measurement was suggested to be a clinically relevant and useful tool for early detection of canine DIC.

## Introduction

DIC is a common clinical condition caused by numerous diseases in dogs seen in veterinary practice. Prognosis was generally poor causing high mortality once dogs develop DIC[1, 2]. However, validated early detection of DIC is not available. Diagnostic criteria for DIC in dogs are not yet fully developed or discussed in veterinary literature. One study defined DIC as a condition characterized by abnormal findings in four out of six parameters including platelet count, prothrombin time (PT), fibrinogen-fibrin degradation product (FDP), activated partial thromboplastin time (APTT), plasma fibrinogen level, fibrinogen fibrin degradation product (FDP), and antithrombin activity (AT) [3]. Another study defined DIC as a condition presenting three abnormal findings including thrombocytopenia, increased FDP, and prolonged PT and/or APTT, and DIC suspect when two abnormal findings were recognized[4]. Definitions of DIC can be found in other studies, however, none of them provide validated evidence for DIC criteria [5–7].

There is no gold standard for diagnosing DIC in humans, however, diagnosis criteria and early detection for DIC have been actively discussed[8–10]. Terminology such as DIC preparatory state, Pre-DIC and impending DIC have been used to segregate conditions before definitive DIC diagnosis to facilitate initiating early treatment for DIC in humans. There is no clear definition for each state, and it is considered DIC should follow preliminary stages such as DIC preparatory state, Pre-DIC or impending DIC before a patient is diagnosed with definitive DIC. It is speculated that dogs also develop Pre-DIC status before DIC, and some advanced techniques for humans are potentially effective in veterinary settings including detection of DIC.

Thrombin is released after the prothrombin complex combines with calcium and phospholipid surface and is intended to create a local clot. Prothrombotic terminate when fibrinogen is converted to fibrin by action of thrombin followed by formation of stabilized fibrin (thrombus) by action of FXIII (blood coagulation factor XIII). Any thrombin that diffuses away from the damaged endothelium will be rapidly inhibited by AT. Excess production of thrombin is major cause of DIC. Therefore, inhibitory reaction of thrombin by AT is activated as a biological defense reaction in the face of DIC, and TAT is generated as a result of series of these reactions. TAT complex is a molecular complex composed of thrombin and AT, a primary thrombin inhibitor, in a 1:1 ratio[11, 12]. Increased TAT indicate excess thrombin production and serve as a marker reflecting prothrombotic status.

Increased thrombin levels signify activation of the blood coagulation cascade. However, thrombin levels cannot be measured directly due to extremely short half-life in blood, whereas TAT has a half-life of 3 to 15 minutes, enabling direct measurement[11]. Consequently, TAT could be used to indirectly assess thrombin generation as a molecular marker of activated coagulation and reflect ongoing condition of DIC. TAT measurement has recently been used in humans for diagnosis and assessment of treatment for disorders including DIC, deep vein thrombosis, and pulmonary thromboembolism[9, 12–19]. TAT is considered to be an effective marker that can detect prothrombotic status earlier than other laboratory testing. TAT is expected to be an important benchmark for early diagnosis for DIC in humans, and DIC could be excluded when TAT level was in normal range [9, 11].

In veterinary medicine, there are a few publications related to TAT measurement. One report suggested validity of TAT in assessing prothrombotic in dogs[20, 21]. Other reports included using TAT as a marker of prothrombotic in canine Cushing’s syndrome, and indicated increased TAT level in dogs with malignant tumors [20, 22]. However, no reports are available evaluating validity of in-house TAT measurement for diagnosis of canine DIC to date.

Previous reports on TAT measurements for dogs were based on EIA methods using human antibodies[20–25]. TAT measurements by EIA methods involve complicated procedures and require prolonged time to yield test results, which make it difficult to reflect the results for rapid treatment at clinical settings. Therefore, it is not common to measure TAT at veterinary hospitals to evaluate prothrombotic. Fukuoka et al reported canine TAT was measurable using the same instrument used in the present study, utilizing Chemi Luminescent Enzyme Assay (CLEIA) by demonstrating dilution test[26]. The measurement system is simple, and the results are available within 30 minutes, and could be reflected to the treatment protocol immediately, whereas conventional EIA takes time to obtain results and may delay initiating early treatment. If this TAT measurement system for dogs could be as useful as for humans, it would be beneficial in clinical setting allowing early detection, treatment, and effective management for clinical conditions such as DIC.

In the present study, TAT levels in healthy dogs and diseased dogs were measured by using a real-time in-house TAT assay device, originally developed for humans, and canine reference interval was determined. The validity of using in-house TAT measurements for diagnoses of DIC was also evaluated.

## Materials and methods

### Study population

A case-control study was performed with dogs admitted to Saitama Animal Medical Center from September 2014 to March 2016. Dogs presented for either health check or neuter without any clinical condition served as controls (group one). Dogs admitted with clinical conditions which received coagulation-fibrinolysis tests were included as cases and were further divided into five groups (groups two to six) depending on the number of abnormal findings observed in coagulation-fibrinolysis tests. Group two included dogs with no abnormal finding observed in any of the six tests for coagulation-fibrinolysis; group three included dogs that showed one abnormal finding; group four included dogs that showed two abnormal findings; group five included dogs that showed three abnormal findings; and group six included dogs that showed four or more abnormal findings.

In this study, groups four and five that presented two to three abnormal findings out of six parameters in coagulation-fibrinolysis testing were defined as DIC preparatory state (Pre-DIC), and group six that presented four or more abnormal findings out of six parameters in coagulation-fibrinolysis testing was defined as DIC.

### Laboratory tests

Complete blood count (CBC), blood chemistry screening, chest X-ray, abdominal ultrasonography, blood pressure, electrocardiography, urinalysis, fecal examination, free thyroxine, and TAT measurements were performed for the dogs admitted for health checks. CBC, serum biochemical analysis, and TAT measurement were performed for dogs admitted for neutering. Dogs with any abnormal finding other than TAT were excluded from the study. Dogs admitted with clinical conditions were tested for platelet count, PT, APTT, FIB, FDP, AT activity, and TAT, in addition to other clinically relevant examinations such as X-rays, abdominal ultrasound, CTs and MRIs. Dogs that did not have results for the seven measurements stated above or those that were diagnosed with immune-mediated thrombocytopenia were excluded from the study.

### Coagulation-fibrinolysis tests

The Hematology analyzer MEK6558 (Nihon Koden, Tokyo, Japan) was used to measure platelet count, and the Sysmex CA-600 (Sysmex, Hyogo, Japan) was used to measure PT, APTT, FIB, FDP and AT activity. The Pathfast (LSI Medience Corporation, Tokyo, Japan) was used to measure TAT level in 0.1 ml blood plasma.

Abnormal findings in coagulation-fibrinolysis tests were defined as follows: Platelet count < 200×10^3^μ/l; PT prolongation > 25% (> 12 seconds); APTT prolongation > 25% (> 25.27 seconds); FIB < 178 mg/dL; FDP > 10μg/mL; and AT activity < 90%.

The cut-off value of <200x10^3^/μL for number of platelets was used in this study to increase detection sensitivity for DIC and to enhance early detection of DIC since temporal reduction rate was more important by serial measurement in humans rather than one-time measurement. In addition, Carr et al also used platelets number <200x10^3^/μL to define thrombocytopenia in DIC diagnostic criteria[4]. Other reports cited <150x10^3^/μL or <179000, however, supportive evidence was not provided[3,5,6].

The lower limit of fibrinogen in normal dogs provided by the manufacturer of the instrument in this study was 178 mg/dL (reference range 178–393 mg/dL). The reference range for normal dogs was different from other reports since the instrument used was different.

### Statistical analysis

Physiologic, hematologic, and other continuous data were assessed for normality of distribution with the Shapiro-Wilk test. Normally distributed continuous variables are reported as mean±SD, and non-normally distributed continuous variables are reported as median (range). The continuous data for TAT measurements were compared between the groups using the Kruskal-Wallis test and simple linear regression model. Reference interval for TAT measurements was determined by using robust method[27]. Receiver Operating Characteristic (ROC) curve was created to determine cut-off value for pre-DIC. The Kruskal-Wallis test was used to analyze 28-day survival rates among the groups. MedCalc Statistical Software version 17.9.2 (MedCalc Software bvba, Ostend, Belgium) was used to determine the reference interval for TAT. STATA (StataCorp. 2015. Stata Statistical Software: Release 14. College Station, TX) was used to perform the Kruskal-Wallis test, simple linear regression model and ROC curve analysis. For statistical estimation and inferences, two-sided hypotheses and tests were used with a 5% significance level.

### Ethics statement

This was an observational study using patient dogs at an animal hospital. TAT levels were measured in blood samples already collected for routine diagnostic laboratory testing requested by owners as part of the patient care, and the dogs were not subjected to any additional blood collections or invasive procedures for the purpose of this research. All owners were informed of the research objectives and provided their consent prior to study enrollment. All dogs were treated and evaluated by board-certified specialists at the hospital and provided with customary high standards of medical care and welfare.

## Results and discussion

A total of 394 dogs were included in the study. Control dogs in group one included 137 dogs that were admitted to the hospital for health check or neuter; of those, 35 were excluded due to abnormalities found in laboratory tests. Sixty-five dogs were included in group two, 78 dogs were included in group three, 65 dogs were included in group four, 29 dogs were included in group five and 20 dogs were included in group six. Observed characteristics of the dogs included in the study were described in Table 1.

**Table 1:** Characteristics of the dogs admitted to the Saitama Animal Medical Center from September 2014 to March 2016.

Variables	Group1(n = 102)	Group 2(n = 65)	Group 3(n = 78)	Group 4(n = 65)	Group 5(n = 29)	Group 6(n = 20)
Sex						
Male	24	15	18	15	7	8
Neutered male	24	21	23	22	6	8
Female	25	8	18	8	8	1
Spayed female	32	21	19	20	8	3
Median age(range)	5.5(0.5–15)	11(0.75–16)	11(1–16)	10(0.42–14)	9(1–18)	8(1–15)
BW (kg) (range)	5.2(1.7–59)	7.4(1.6–29.3)	7.6(1.5–35.5)	7.7(1.9–42.5)	5.9(1.5–58)	13.26(3.3–42.5)

Median TAT levels (range) were 0.06 ng/ml (0.019–0.49), 0.11 ng/ml (0.03–0.88), 0.29 ng/ml (0.004–3.8), 2.59 ng/ml (0.034–51.8), 3.85 ng/ml (0.29–35.0) and 9.76 ng/ml (0.86–75.4) for group 1, 2, 3, 4, 5 and 6, respectively (p = 0.0001). There was no significant difference in TAT levels between the control group, group two (p = 0.916) and three (p = 0.772); however, group four (p = 0.012), group five(p<0.0001), and group six were (p<0.0001) higher than the control group (Table 2).

**Table 2:** Simple linear regression model for TAT level in dogs admitted to the Saitama Animal Medical Center from September 2014 to March 2016 (n = 359).

Variable	Coefficient	95% CI	p-value
Group 1 (n = 102)	Reference		
Group 2 (n = 65)	0.11	-2.02–2.25	0.916
Group 3 (n = 78)	0.28	-1.78–2.34	0.772
Group 4 (n = 65)	2.57	0.40–4.75	0.012
Group 5 (n = 29)	5.18	2.30–8.07	<0.0001
Group 6 (n = 20)	15.29	11.94–18.64	<0.0001

**Fig 1:**
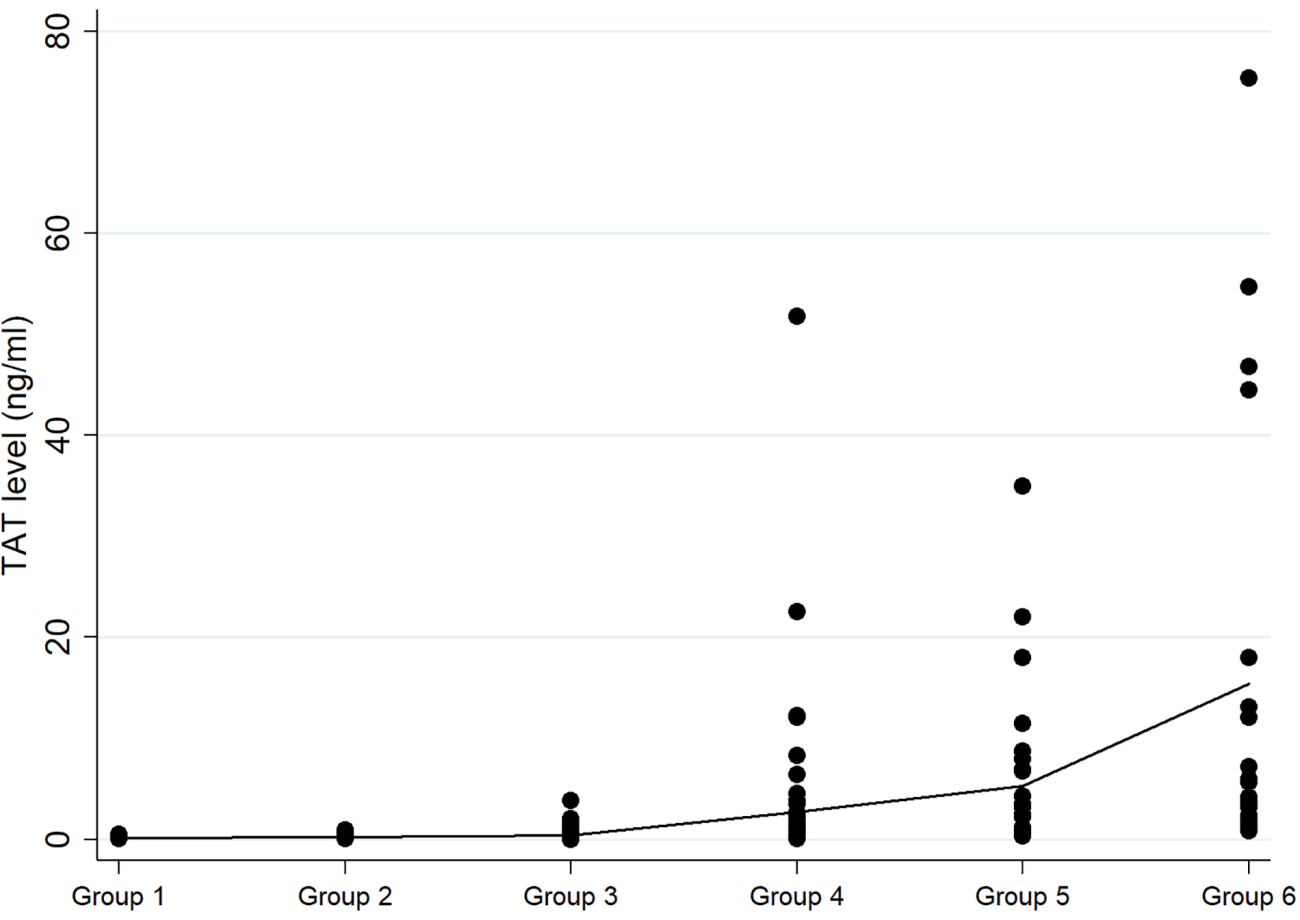
TAT levels for the dogs (Group 1 to 6) admitted to the Saitama Animal Medical Center from September 2014 to March 2016 (n = 359).

**Fig 2:**
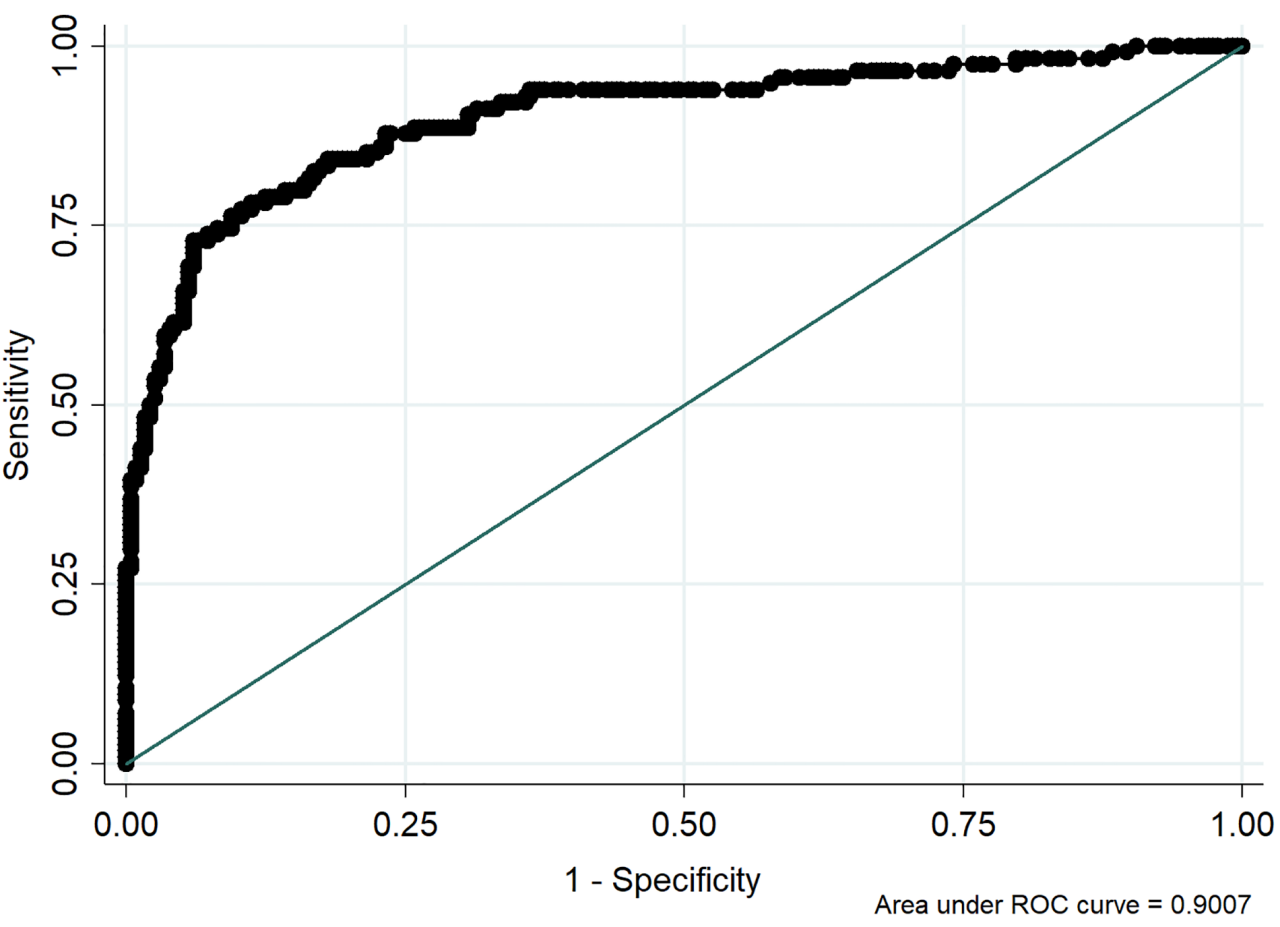
ROC curve for Pre-DIC cut-off by TAT level (n = 346) in dogs admitted to the Saitama Animal Medical Center from September 2014 to March 2016. The cut-off TAT level was 0.35 ng/ml (sensitivity 84.2, specificity 81.9%, AUC 82.7%).

The association of TAT among the groups was described in Fig 1. The reference interval of TAT level in healthy dogs was <0.25 ng/ml. Pre-DIC cut-off level of TAT was determined as 0.35 ng/ml (sensitivity 84.2%, specificity 81.9%, AUC 82.7%) by ROC curve (Fig 2).

Twenty-eight days survival rates were, 97.8% (45/46), 91.4% (64/70), 75.8% (47/62), 71.4% (20/28) and, 50% (9/18) for groups one, two, three, four, five and six, respectively (p < 0.0001).

In human medicine, measurement of TAT has recently been utilized for detection of DIC, not only as a diagnostic indicator but also as an important tool for monitoring treatment. On the other hand, there have been no reports on investigating validity of TAT measurement for diagnosis of canine DIC to date and this study was the first report to evaluate in-house measurement of TAT for diagnosis of DIC. In the present study, TAT level was positively associated with severity of DIC. There was no significant difference among groups one (clinically healthy dogs), two (diseased dogs without abnormality in the coagulation-fibrinolysis tests) and three (one abnormal finding in the coagulation-fibrinolysis tests), however, TAT level was 2.57 ng/ml higher in group four, 5.18 ng/ml higher in group five, and 15.29 ng/ml higher in group six compared to the control group. These results suggest that TAT measurement would be validated and useful in detecting and monitoring ongoing prothrombotic; i.e. Pre-DIC and DIC, in dogs as in humans.

Reference interval for TAT in this study was found to be < 0.25 ng/ml. Human reference range in previous report was <3 ng/ml[11] and normal canine TAT level was considered to be lower than human. The number of reports on the reference level of TAT by conventional EIA method is limited. One reported the median TAT level for normal dogs (n = 16) was 0.5ng/ml[1], whereas another reported that the mean TAT value was 2.0 ng/ml in 23 normal dogs[2]. It is known that TAT level measured by CLEIA correlated with the results from conventional EIA in humans. Correlation in dogs had only been presented orally in Japan, and published data is not available. Further research is required to validate correlation of TAT levels by EIA method and CLEIA method in dogs.

One study used the same testing system as the present study and validated the measurement of canine TAT by dilution test[26]. However, they did not evaluate the reference range of TAT for normal dogs. The median of TAT level for 102 normal dogs in the present study was 0.06ng/ml (range 0.019–0.49).

The cut-off TAT value for pre-DIC was found to be 0.35 ng/ml. This value was clinically relevant and could be used as one criterion to implement vigorous treatment for DIC or prospect prognosis. The cut-off TAT level for Pre-DIC in the present study was based on a limited number of diseased animals and warrants further investigation involving a larger sample size in the future.

Diagnosis of canine DIC was generally based on assessment of parameters including thrombocytopenia, prolonged PT and APTT, decreased FIB, increased FDP, and decreased AT activity, in addition to underlying diseases[3–7, 28–30]. Increased FDP could be from breakdown of fibrinogen, fibrin monomer or cross-linked fibrin. These parameters are not effective for early detection or definitive diagnosis of canine DIC, since they do not reflect active coagulation. Although decreased platelet count may reflect platelet consumption caused by active coagulation, many conditions other than DIC could also induce thrombocytopenia[9, 31], such as sepsis where low platelet counts were caused by gram-negative bacteria[9]. In chronic liver disease, reduced thrombopoietin production minimized platelet production[9] and immune-mediated thrombocytopenia also caused a reduction in the number of platelets, therefore, platelet count could not be simply used as a diagnostic criterion for DIC in these diseases [9, 11]. Therefore, being able to measure TAT on site is extremely valuable for rapid and accurate diagnoses for DIC.

Prolonged PT and APTT resulting from depletion of blood coagulation factors were caused by excessive activation of coagulation. However, such changes in PT and APTT would not be manifested until the coagulation factors dropped below 30% of the normal range and this time lag made early detection of coagulation status difficult [9, 31]. Congenital or acquired deficiencies of coagulation factors other than by DIC such as hemophilia, factor VII deficiency, and warfarin poisoning, could also develop prolonged PT and APTT[9, 31]. Low FIB and high FDP levels may be caused by hyperfibrinolysis, however, manifestations are generally mild in DIC with suppressed fibrinolysis[9, 11]. Non-DIC cases such as thrombosis, pleural effusion, ascites, and hematoma can also increase FDP that enter the bloodstream and elevate FDP level in the absence of DIC[9, 11]. Elevated FIB level in the face of inflammation indicated that FIB level would not decrease by DIC[1, 9, 31]. Abnormal TAT level is recognized only as a result of prothrombotic (DIC or thrombosis other than DIC), whereas other parameters in coagulation-fibrinolysis testing show abnormal values in the face of various conditions other than DIC and are also influenced by different types of DIC (whether fibrinolysis is suppressed or increased). It is feasible to exclude DIC if TAT level is in normal range even with any underlying disease, and if TAT is elevated, thrombotic tendency could be strongly suspected.

TAT is formed by binding AT to thrombin in a ratio of 1:1. DIC induces excessive production of thrombin that causes binding of AT to thrombin, therefore, inhibits AT activity. However, AT was originally found more than thrombin in the body; thus, active coagulation alone would not cause substantial reduction of AT activity[11]. In fact, inhibited AT activity was generally caused by conditions other than DIC such as liver failure and sepsis where inflammation stimulated not only loss of AT from bloodstream, but also degradation of AT by granulocyte elastase or hypoalbuminemia causing reduced AT activity in relation to albumin[9].

In humans, increased TAT level is known to reflect increased thrombin level and prothrombotic, and has been established as a key marker for DIC[32]. DIC, Pre-DIC, and thromboembolism are all considered to be common cause of elevated TAT level[33–36]. In addition, some studies indicated TAT was a more sensitive marker for coagulation than FDP or other fibrinolytic markers in diagnosing DIC[34, 36]. TAT in normal range is considered to be a robust criterion to exclude DIC[9, 11].

In veterinary medicine, using TAT remains uncommon and there is a limited number of reports on demonstrating TAT as an effective coagulation marker. McMichael et al. employed TAT and thromboelastography to assess prothrombotic in dogs with blastomycosis[23]. TAT level was found to be significantly higher in diseased dogs than in control dogs, consistent with TEG results indicating prothrombotic in diseased dogs.[23] In another study, dogs with chronic CHF appeared to develop prothrombotic and TAT level was significantly higher in dogs with CHF compared to healthy dogs[24]. In the present study, none of the study animals had blastomycosis or CHF. Administration of human immunoglobulin G preparation was reported to produce prothrombotic in dogs[25]. Elevated TAT and FDP were recognized as coagulation markers to detect prothrombotic in dogs that were administered human immunoglobulin G[25]. However, none of the dogs were administered human immunoglobulin G in the present study. All the study animals in the present study that indicated elevated TAT level were due to DIC.

Prognosis of DIC was known to be poor in dogs and cats [5, 30, 37]. This was evident in 28-day survival rates in the present study; in dogs with two and three abnormal values in coagulation-fibrinolysis test, the 28-day survival rates were 75.8% and 71.4%, respectively, whereas the 28-day survival rate was only 50% in dogs with four or more abnormal values in coagulation-fibrinolysis test. Our results also showed TAT level tended to increase with higher number of abnormal results in coagulation-fibrinolysis tests, suggesting that TAT may serve as an effective indicator for determining severity of DIC. Assessment of TAT level is valuable when treating dogs with a disease suspected to be associated with prothrombotic. It is worthwhile to suggest TAT measurement should be included in the diagnostic criteria for canine DIC.

There is no validated gold standard for diagnosis of DIC in the veterinary medicine, therefore, diagnosis of canine DIC was rather imprecise and general compared to human medicine. In addition, platelet count, PT, APTT, FIB, FDP, and AT activity would not provide enough information for early detection of DIC, since these abnormal parameters did not reflect ongoing prothrombotic directly but only the result of prothrombotic[9, 11]. However, TAT measurement indicated production of thrombin indirectly, therefore, reflected ongoing prothrombotic [1, 11, 12]. Thrombelastography (TEG) and Rotational Thromboelastrometry (ROTEM) were the tests that could evaluate not only prothrombotic but also hyperfibrinolysis and hypocoagulation [38, 39], and several studies used these tests to evaluate blood coagulation in dogs[30, 38–50]. Wiinberg et al used TEG to evaluate 50 dogs with DIC and demonstrated the validity of diagnosability of DIC and mortality was higher in dogs with prothrombotic than hypocoagualtion [30]. However, there are limitations in ROTEM and TEG for evaluating DIC, since prothrombotic results in ROTEM and TEG is affected by hematocrit (HTC) [44, 46, 51–55]. In addition, because of the intrinsic nature of testing system, it is difficult to differentiate prothrombotic from DIC in the presence of thrombocytopenia and decreased fibrinogen. On the other hand, TAT measurement is superior in terms of evaluating DIC because TAT is not affected by HCT and other parameters.

## Conclusions

Previous studies and our study indicated in-house TAT measurement was beneficial for dogs in detecting prothrombotic including DIC as in humans. In-house TAT measurement yields rapid results and may improve diagnostic accuracy for DIC when used in conjunction with conventional diagnostic testing such as D-dimer, FDP and/or platelet counts.

## Supporting information

S1 Data Raw data collected at the study site. (XLSX)

S1 Supplemental file Underlying disease observed in each group of dogs in the study. (DOCX)
