# Supplementary material for: Thrombin-antithrombin complex measurement using a point-of-care testing device for diagnosis of disseminated intravascular coagulation in dogs
Source: PLoS One. 2018 Oct 10;13(10):e0205511. doi: 10.1371/journal.pone.0205511 (PMC6179255; doi:10.1371/journal.pone.0205511)
Supplement: S1 Supplemental file — (DOCX) [file pone.0205511.s002.docx]

| Group 2 | Disease | Number of dogs |
| --- | --- | --- |
| Non-neoplastic disease  (n＝18) |  |  |
|  | Chronic enterocolitis | 4 |
|  | Rectal polyp | 2 |
|  | Proctocele | 1 |
|  | Fibrous polyp in the auditory cana | 1 |
|  | Vaginal polyp | 1 |
|  | Stomach twisting | 1 |
|  | Chronic uveitis | 1 |
|  | Acute pancreatitis | 1 |
|  | Cholecystitis | 1 |
|  | Pyometra | 1 |
|  | Diabetes | 1 |
|  | Cushing's syndrome | 1 |
|  | Pelvic fracture | 1 |
|  | Radius fracture | 1 |
| Malignant tumor (n＝24) | Mast cell tumor | 3 |
|  | Osteosarcoma | 2 |
|  | Bladder transitional cell carcinoma | 2 |
|  | Intraoral squamous cell carcinoma | 2 |
|  | Bowel adenocarcinoma | 1 |
|  | Undifferentiated sarcoma | 1 |
|  | Malignant mesothelioma | 1 |
|  | Gastrointestinal interstitial cells tumor | 1 |
|  | Lymphoma | 1 |
|  | Multiple myeloma | 1 |
|  | Liposarcoma | 1 |
|  | Soft tissue sarcomas | 1 |
|  | Histiocytic sarcoma | 1 |
|  | Infiltrating lipoma | 1 |
|  | Malignant thymoma | 1 |
|  | Pulmonary adenocarcinoma | 1 |
|  | Splenic angiosarcoma | 1 |
|  | Exocrine pancreas cancer | 1 |
|  | Mammary adenocarcinoma | 1 |
| Benign tumor (n＝7) | Perianal adenoma | 2 |
|  | Mammary adenoma | 2 |
|  | Melanoma | 2 |
|  | Hemangioma | 1 |
| Others（n＝6) | Mandibular mass | 1 |
|  | Vestibular disorder | 1 |
|  | Splenic mass | 1 |
|  | Ovarian tumor suspicion | 1 |
| Group 3 (n＝78) |  |  |
| Non-neoplastic lesion (n＝34) | Pyometra | 9 |
|  | Chronic enterocolitis | 4 |
|  | Acute pancreatitis | 4 |
|  | Immune-mediated hemolytic anemia | 3 |
|  | Ileus | 2 |
|  | Polyarthritis | 2 |
|  | Lymphangiectasia | 2 |
|  | Chronic uveitis | 1 |
|  | Trauma | 1 |
|  | Chronic renal failure | 1 |
|  | Dentigerous cyst | 1 |
|  | Root abscess | 1 |
|  | Chronic pancreatitis | 1 |
|  | Gallbladder cystomyxoma | 1 |
|  | Bladder stone | 1 |
| Malignant tumor (n＝34) | Mast cell tumor | 8 |
|  | Lymphoma | 6 |
|  | Maxillary undifferentiated sarcoma | 3 |
|  | Intraoral malignant melanoma | 3 |
|  | Mammary adenocarcinoma | 3 |
|  | Brain tumor | 2 |
|  | Hepatocellular carcinoma | 2 |
|  | Thyroid carcinoma | 1 |
|  | Ocular malignant melanoma | 1 |
|  | Osteosarcoma | 1 |
|  | Malignant mesothelioma | 1 |
|  | Histiocytic sarcoma | 1 |
|  | Soft tissue sarcoma | 1 |
|  | Mandibular malignant tumor | 1 |
| Benign tumor（n＝3) | Mammary adenoma | 3 |
| Others（n＝7) | Adrenal enlargement | 3 |
|  | Intraabdominal mass | 1 |
|  | Bone tumor suspicion | 1 |
|  | Liver mass | 1 |
|  | Unidentified fever | 1 |
| Group 4 |  |  |
| Non-neoplastic lesion (n＝27) | Gallbladder cystomyxoma | 5 |
|  | Chronic enterocolitis | 4 |
|  | Immune-mediated hemolytic anemia | 2 |
|  | Liver microvessel hypoplasia | 2 |
|  | Trauma | 1 |
|  | Hyperadrenocorticism | 1 |
|  | Splenic hematoma | 1 |
|  | Polyarthritis | 1 |
|  | Perineal hernia | 1 |
|  | Systemic circulation portal shunt | 1 |
|  | Pneumonia | 1 |
|  | Splenic nodular hyperplasia | 1 |
|  | Acute pancreatitis | 1 |
|  | Congenital renal hypoplasia | 1 |
|  | Ventral hernia | 1 |
|  | Extradural haematoma | 1 |
| Malignant tumor (n＝26) | Lymphoma | 10 |
|  | Histiocytic sarcoma | 3 |
|  | Mammary adenocarcinoma | 2 |
|  | Splenic liposarcoma | 1 |
|  | Splenic angiosarcoma | 1 |
|  | Splenic undifferentiated sarcoma | 1 |
|  | Renal angiosarcoma | 1 |
|  | Intranasal adenocarcinoma | 1 |
|  | Liver neuroendocrine tumor | 1 |
|  | Intraoral squamous cell carcinoma | 1 |
|  | Rectal leiomyosarcoma | 1 |
|  | Hepatocellular carcinoma | 1 |
|  | Thyroid carcinoma | 1 |
| Benign tumor (n＝8) | Mammary adenoma | 3 |
|  | Hepatocellular adenoma | 1 |
|  | Melanoma | 1 |
|  | Melanoma | 1 |
|  | Fibromatous epulis | 1 |
| Others（n＝4) | Adrenal enlargement | 2 |
|  | Anterior mediastinum mass | 1 |
|  | Splenic mass | 1 |
| Group 5 |  |  |
| Non-neoplastic lesion (n＝22) | Splenic hematoma | 3 |
|  | Pyometra | 3 |
|  | Bile duct hepatitis | 3 |
|  | Gallbladder cystomyxoma | 2 |
|  | Acute pancreatitis | 2 |
|  | Sepsis | 2 |
|  | Lymphadenitis | 1 |
|  | Immune-mediated hemolytic anemia | 1 |
|  | Pyloric stenosis | 1 |
|  | Systemic circulation portal shunt | 1 |
|  | Right heart failure | 1 |
|  | Addison crisis | 1 |
|  | Chronic enterocolitis | 1 |
| Malignant tumor (n＝7) | Splenic angiosarcoma | 3 |
|  | Lymphoma | 1 |
|  | Pulmonary carcinoma | 1 |
|  | Soft tissue sarcomas | 1 |
|  | Histiocytic sarcoma | 1 |
| Group 6 |  |  |
| Non-neoplastic lesion (n＝9) | Sepsis | 3 |
|  | Splenic hematoma | 2 |
|  | Prostatitis | 1 |
|  | Spinal cord injury (traffic accident) | 1 |
|  | Dirofilaria immitis symptom | 1 |
|  | Immune-mediated hemolytic anemia | 1 |
| Malignant tumor (n＝10) | Splenic angiosarcoma | 3 |
|  | Lymphoma | 2 |
|  | Splenic undifferentiated sarcoma | 1 |
|  | Prostatic carcinoma | 1 |
|  | Intranasal adenocarcinoma | 1 |
|  | Gastric adenocarcinoma | 1 |
|  | Histiocytic sarcoma | 1 |
| Others（n＝1) | Unidentified fever | 1 |
